# When Weakness Persists Beyond Its Apparent Cause: A Case Report of Myasthenia Gravis

**DOI:** 10.7759/cureus.116581

**Published:** 2026-09-20

**Authors:** Noor Ehsan, Hateem Ahmed, Mahnoor Iqbal, Maryam Arshad, Muhammad Imran

**Affiliations:** 1 Internal Medicine, Gulab Devi Teaching Hospital, Lahore, PAK

**Keywords:** generalised weakness, hypokalemia, myasthenia gravis, ptosis, pyridostigmine

## Abstract

Myasthenia gravis (MG) is an autoimmune disorder of the neuromuscular junction characterised by fluctuating, fatigable skeletal muscle weakness. Its overlap with reversible causes of weakness such as hypokalemia can easily delay recognition. We report the case of a 40-year-old man who presented with one week of profuse diarrhea and vomiting, along with two days of generalized limb weakness. Investigations revealed hypokalemia and hypomagnesemia, and his limb weakness partially improved after fluid and electrolyte correction, which raised suspicion of an additional underlying cause contributing to the persistent weakness. He was noted to have bilateral ptosis, and further history uncovered three months of fatigable weakness that worsened towards the end of the day, with slurred nasal speech, difficulty chewing, and nasal regurgitation. Examination showed a positive curtain sign, bilateral ptosis that improved with ice pack test, and fatigable proximal limb weakness with preserved reflexes. Anti-acetylcholine receptor antibodies were strongly positive, and contrast-enhanced computed tomography (CT) scan of the chest revealed the presence of thymoma in the anterior mediastinum. He responded well to pyridostigmine and oral prednisone. Thymectomy was subsequently planned. This case is a reminder that weakness outlasting its apparent cause warrants a neurological evaluation, since a concurrent electrolyte disturbance can readily mask an underlying myasthenic process.

## Introduction

Myasthenia gravis (MG) is an autoimmune disorder of the neuromuscular junction that causes fluctuating and fatigable skeletal muscle weakness due to impaired neuromuscular transmission [1,2]. In most patients, antibodies are directed against postsynaptic acetylcholine receptors (AChRs), affecting normal neuromuscular transmission [1,3,4]. The disease may be limited to the ocular muscles or may progress to generalized MG involving the bulbar, limb, neck, and respiratory muscles [2,3]. Ocular symptoms commonly include ptosis and diplopia, while bulbar involvement may cause dysarthria, dysphagia, fatigable chewing, facial weakness, and nasal regurgitation [1,2,4]. A typical feature is worsening of weakness with activity and improvement with rest [1,2,3]. Severe respiratory muscle weakness can lead to myasthenic crisis, a potentially life-threatening complication [1,3,5]. MG is also associated with thymic abnormalities, particularly thymoma, which is found in approximately 10-15% of patients with MG and is more commonly associated with AChR-antibody-positive disease [1,6].

Hypokalemia is an important and potentially reversible cause of muscle weakness and may occur due to potassium loss from prolonged diarrhea and vomiting [7]. Severe hypokalemia can cause generalized or predominantly proximal muscle weakness and, in some cases, hypokalemic paralysis [7]. Although bulbar involvement is uncommon, rare cases of hypokalemic paralysis have been reported with dysarthria and dysphagia, with improvement following potassium correction [8]. This clinical overlap can make it difficult to distinguish hypokalemic weakness from an underlying neuromuscular disorder [7,8].

We present the case of a 40-year-old male patient with significant hypokalemia and generalized weakness that showed partial improvement with electrolyte repletion. The subsequent recognition of fatigable ocular and bulbar symptoms, along with proximal limb weakness, strongly positive anti-AChR antibodies, and thymoma on a computed tomography (CT) scan of the chest confirmed generalized AChR-antibody-positive MG.

## Case presentation

A 40-year-old male patient presented to the outpatient department of a tertiary care hospital with profuse non-bloody diarrhea for one week, with 10-15 watery episodes per day occurring after meals and during fasting, including at night. He also had occasional non-bloody vomiting containing undigested food particles. He consumed water from a local filtration plant. There was no recent history of travel, sick contacts, hospital admission, antibiotic, laxative, or herbal medication use. He reported reduced urine output. At presentation, he was also found to have generalized limb weakness for two days, involving all four limbs approximately simultaneously, with the upper limbs more affected than the lower limbs. There was no ascending pattern of weakness, sensory symptoms, or autonomic dysfunction. He had no shortness of breath, orthopnea, or other respiratory difficulty.

On examination, his blood pressure (BP) was 100/60 mmHg (normal range: systolic BP, 90-119 mmHg; diastolic BP, 60-79 mmHg), pulse was 92 beats/minute and regular (normal range: 60-100 beats/minute), respiratory rate was 15 breaths/minute (normal range: 12-20 breaths/minute), temperature was 98.5°F (normal range: 97-99°F), and oxygen saturation was 96% on room air (normal range: 95-100%). He had reduced skin turgor, a dry tongue, mild lower abdominal tenderness, and hyperactive bowel sounds. Neurological examination showed 3/5 power in the proximal and 4/5 power in the distal muscle groups of the upper limbs, with 4/5 power in both proximal and distal muscle groups of the lower limbs. Muscle tone was normal, deep tendon reflexes were intact, plantars were flexor bilaterally, and there was no sensory deficit. He was not using accessory muscles of respiration. Laboratory investigations showed hypokalemia and hypomagnesemia. The complete metabolic panel and the complete blood count are shown in Table 1.

**Table 1:** Laboratory investigations

Investigation	Patient value	Reference range
Hemoglobin	15.4 g/dL	13 - 18 g/dL
Total leucocyte count	11000 / μL	4000 - 11000 /μL
Platelets	207000 /μL	150000 - 450000 /μL
Serum creatinine	0.8 mg/dL	0.6 - 1.1 mg/dL
Serum potassium	2.7 mmol/L	3.5 - 5.5 mmol/L
Serum sodium	145 mmol/L	135 - 145 mmol/L
Serum magnesium	1.4 mg/dL	1.60 - 2.60 mg/dL
Serum phosphate	2.5 mg/dL	2.5 - 4.5 mg/dL
Serum bicarbonate	21 mEq/L	22 - 26 mEq/L
Serum chloride	108 mEq/L	96 - 106 mEq/L
Serum calcium	9.0 mg/dL	8.8 - 10.2 mg/dL
Serum pH (arterial)	7.36	7.35 - 7.45

ECG showed U waves in the chest leads. Stool analysis was negative for ova and parasites, and a stool culture was subsequently performed, with the results reported below.

He was initially managed with intravenous fluids, potassium and magnesium replacement, and empiric ceftriaxone. His urine output improved following hydration. Vital capacity and maximal inspiratory pressure (MIP) were measured six-hourly and remained within normal limits without a decreasing trend. Once vomiting settled, he was switched to oral rehydration. Stool culture showed no pathogenic growth after 48 hours, and ceftriaxone was discontinued. His potassium and magnesium levels subsequently normalized (Table 2). 

**Table 2:** Laboratory investigations

Investigation	Patient value	Reference range
Serum potassium	3.6 mmol/L	3.5 - 5.5 mmol/L
Serm magnesium	1.7 mg/dL	1.60 - 2.60 mg/dL
Serum bicarbonate	22 mEq/L	22 - 26 mEq/L
Serum chloride	106 mEq/L	96 - 106 mEq/L

Following correction of the electrolyte abnormalities, his power improved partially. Upper limb proximal power partially improved to 4/5 and distal power to 5/5, while lower limb power was 4/5 proximally and 5/5 distally. This suggested that hypokalemia had contributed to his acute limb weakness; however, the weakness did not completely resolve. This prompted consideration of another underlying cause contributing to the persistent weakness.

During evening rounds, bilateral ptosis was noted. On further questioning, he reported generalized weakness and fatigue for approximately three months, with difficulty keeping his eyes open towards the end of the day. He also described slurring of speech and difficulty chewing towards the end of meals, particularly during dinner, occasionally associated with nasal regurgitation of fluids. His symptoms worsened as the day progressed and were better in the morning when he woke up. He had worked as a painter but had recently quit because he could no longer hold his paintbrush above his head while painting ceilings. He was also finding it increasingly difficult to climb stairs. There was no history of respiratory symptoms, sensory disturbance, gait disturbance, or bladder dysfunction. He had no muscle pain, joint pain, rash, or photosensitivity and was not taking any long-term medications. He had a 12.5 pack-year smoking history but no symptoms suggestive of lung malignancy.

Repeat neurological examination showed bilateral ptosis, with Margin Reflex Distance (MRD) 1 of 2 mm in the right eye and 1 mm in the left eye. Ptosis worsened after sustained upward gaze for 60 seconds, with a positive curtain sign, and improved with the ice pack test. His speech had a pronounced nasal quality and became more slurred after counting to 100 in one minute. He had a characteristic vertical smile, with an intact gag reflex. Proximal power in both upper and lower limbs decreased to 3/5 with repeated muscle movement against gravity and resistance. The presence of fatigable ocular, bulbar, and proximal limb weakness with preserved sensation and intact reflexes raised suspicion of myasthenia gravis. Lambert-Eaton myasthenic syndrome was considered less likely because the weakness worsened with sustained activity rather than improving with repeated use, reflexes were preserved, and the workup was negative for an underlying lung malignancy.

AChR antibody levels were strongly positive, confirming acetylcholine receptor antibody-positive myasthenia gravis. His thyroid-stimulating hormone (TSH) level was within normal limits (Table 3).

**Table 3:** Laboratory investigations

Investigation	Patient value	Reference range
Anti-acetylcholine receptor antibody	8.0 nmol/L	Negative: <0.40 nmol/L, Borderline: 0.4 - 0.50 nmol/L, Positive: >0.50 nmol/L
Thyroid-stimulating hormone	1.54 mIU/mL	0.4 - 4.2 mIU/mL

Pyridostigmine was started at 30 mg orally three times daily and gradually increased to 60 mg three times daily, with improvement in his symptoms and ptosis, although ptosis did not completely resolve. MRD 1 improved to 2.5 mm in the right eye and 2 mm in the left eye. Further dose escalation to a total of 240 mg per day resulted in cholinergic excess, including muscle fasciculations, excessive salivation and diarrhea. Pyridostigmine was therefore de-escalated and maintained at 60 mg three times daily. Oral prednisone was started at 10 mg daily and increased by 5 mg every five days to 40 mg daily, with no further worsening of myasthenic symptoms. Bone and gastric protection were added. His ptosis resolved and speech recovered completely with concurrent prednisone and pyridostigmine. A contrast-enhanced CT scan of the chest showed a mass in the anterior mediastinum suggestive of thymoma. There was no evidence of a lung malignancy (Figure 1).

**Figure 1:**
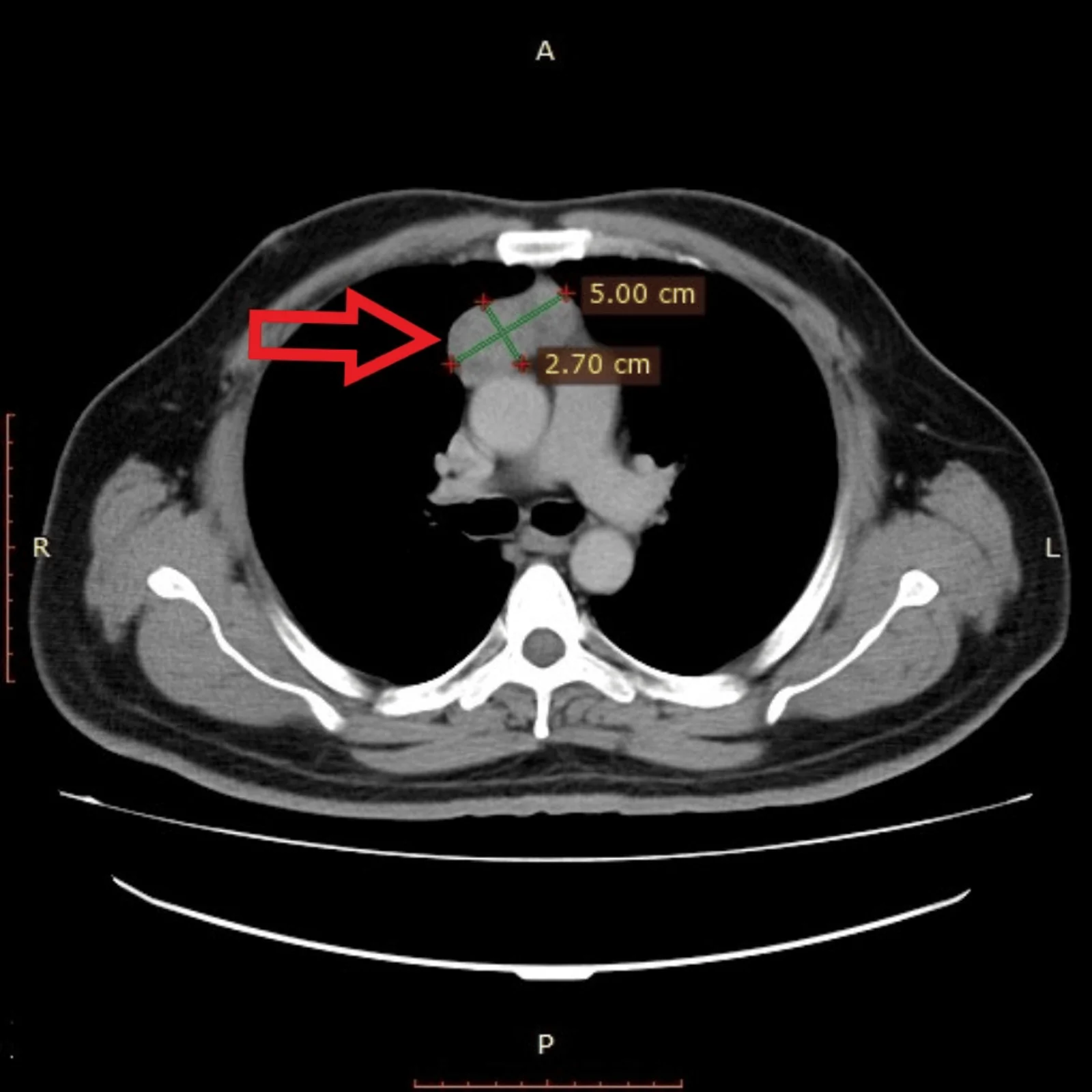
Contrast-enhanced computed tomography (CT) scan of the chest demonstrating the presence of thymoma (marked with the arrow) CT scan of the chest showing a 5.0 × 2.7 × 5.6 cm soft-tissue mass in the anterior mediastinum, suggestive of thymoma, without vascular encasement or mediastinal lymphadenopathy. The lung parenchyma is clear bilaterally.

Once his symptoms were controlled, the thoracic surgery department was consulted for thymectomy. He was established on long-term follow-up in the medicine and neurology outpatient departments, and his symptoms remain well controlled.

## Discussion

MG is a disorder of neuromuscular transmission that can be difficult to recognize when weakness occurs in the presence of another acute illness or a plausible metabolic abnormality [2]. The diagnosis relies largely on recognizing the characteristic pattern of fatigable weakness, particularly when ocular and bulbar muscles are involved. Diurnal variation, worsening with sustained activity, and improvement with rest are important clinical features that help distinguish MG from other causes of generalized weakness [1]. Juel et al. described a similar diagnostic challenge in the evaluation of generalized weakness, emphasizing the importance of considering neuromuscular disorders when the clinical course was not fully explained by an initial diagnosis [2].

MG is classically a disorder of young women, with population-based studies consistently showing female predominance below the age of forty and a second, smaller peak in older men [1,9]. At 40 years of age, this patient did not fit the typical demographic pattern of early-onset disease. The presence of thymoma provides an important clinical context. Thymoma is found in roughly 10-15% of all patients with MG, clusters in the fifth and sixth decades, and carries little of the sex bias seen in autoimmune MG [1]. Current consensus recommendations support contrast-enhanced chest imaging in newly diagnosed MG to assess for thymoma [10,11]. Our patient's demographic profile also warranted chest imaging. In this patient, contrast-enhanced CT scan of the chest subsequently demonstrated an anterior mediastinal thymoma. 

MG is broadly classified into ocular and generalised forms; around half of patients begin with purely ocular symptoms, but a significant proportion of these generalise within two to three years [1,3]. Our patient had fatigable ptosis together with bulbar features and proximal limb weakness; this placed him in the generalised, bulbar-predominant category, Myasthenia Gravis Foundation of America (MGFA) clinical classification class IIIb [12]. Bulbar involvement matters because it carries a higher risk of progression to myasthenic crisis, and it is one reason we did not treat him with pyridostigmine alone [1,3,13]. The strongly positive anti-AChR antibody confirmed the diagnosis. It distinguished him from the muscle-specific kinase (MuSK), low-density lipoprotein receptor-related protein 4 (LRP4), and seronegative subgroups, which behave and respond differently [13].

Pyridostigmine was used for symptomatic treatment by increasing acetylcholine availability at the neuromuscular junction through acetylcholinesterase inhibition [1,4]. Higher doses of pyridostigmine can result in cholinergic excess. Muscarinic overdrive may lead to excess salivation, cramping and sweating, while nicotinic overdrive can cause fasciculations and, perversely, worsen weakness through depolarisation blockade [4]. Cholinergic over-treatment and inadequate treatment of MG may present similarly, as both can cause worsening weakness; consequently, escalating pyridostigmine in response to worsening weakness may precipitate iatrogenic cholinergic crisis [4]. Our patient developed signs of cholinergic excess as the dose of pyridostigmine was increased. The distinction was supported by the presence of muscarinic symptoms and by their temporal association with pyridostigmine dose escalation. Hence, the dose of pyridostigmine was reduced, and immunotherapy started, as supported by literature [4,5].

Corticosteroids are effective as immunotherapy in MG. However, a substantial proportion of patients experience a transient worsening of weakness in the first one to two weeks of treatment, with a minority progressing to myasthenic crisis [5,14]. The phenomenon is thought to reflect an early surge of autoantibody-mediated attack on the already fragile endplate before the immunosuppressive effects of the steroid take hold. This is why the modern, guideline-endorsed approach is to start low and climb slowly, reserving high starting doses for patients with severe disease or with progressive symptoms, who can be closely monitored in the inpatient setting and supported with plasmapheresis or intravenous immunoglobulins [5,10,11]. Long-term corticosteroid use carries important adverse effects, including weight gain, glucose intolerance, and osteoporosis. Steroid-sparing therapy is indicated in patients who suffer from such adverse effects, who fail to respond to steroids, or who show recurrence of symptoms when steroids are tapered [4,10,11]. Azathioprine is a commonly used steroid-sparing immunosuppressant for such patients [10,11,15]. 

The thymus has a central role in the pathogenesis of AChR antibody-positive MG. Thymic myoid cells display the AChR, thymic antigen-presenting cells present it to the T cells, and the hyperplastic thymus even builds ectopic germinal centres that manufacture anti-AChR antibody on site [6]. In thymoma-associated MG, the tumour epithelium fails negative selection, exports autoreactive T cells, and cannot make regulatory T cells properly, which is why thymoma-associated MG is often more severe and is enriched with additional antibodies against titin and the ryanodine receptor [16]. The randomised trial of thymectomy in myasthenia gravis (MGTX trial) proved that thymectomy decreases steroid dose and azathioprine requirement, reduces hospitalisation rates, and distress associated with symptoms even in non-thymomatous, anti-AChR-antibody-positive patients with MG [17]. Thymoma is a potentially malignant epithelial neoplasm staged and classified by WHO criteria. Thymectomy is indicated from an oncological point of view, independent of its potential benefits for MG [18,19]. Perioperative stabilization is particularly important because patients with active MG are at risk of postoperative respiratory weakness and prolonged ventilation. Consensus recommendations support optimization of neuromuscular function before thymectomy, with plasma exchange or intravenous immunoglobulin considered when rapid preoperative improvement is required [10,11,17]. Patients with thymoma also had an increased risk of other autoimmune and malignant conditions, making continued follow-up important beyond treatment of the thymoma and control of neuromuscular symptoms [19].

The main diagnostic challenge was the presence of hypokalemia as a plausible explanation for the acute weakness. Although muscle power showed partial improvement after potassium correction, persistent limb weakness with fatigable ocular and bulbar symptoms led to the diagnosis of MG. The subsequent development of cholinergic excess highlights the narrow therapeutic margin of pyridostigmine. Repetitive nerve stimulation and single-fiber electromyography were not performed, as the characteristic clinical history and examination findings, together with strongly positive anti-AChR antibody titers, were considered sufficient to establish the diagnosis. As a single case, this report cannot establish causality but highlights important diagnostic and therapeutic considerations in generalized AChR-antibody-positive MG.

## Conclusions

This case highlights that improvement after correction of a reversible metabolic abnormality does not exclude a concurrent neuromuscular disorder. Persistent or fluctuating weakness despite normalization of electrolyte abnormalities should prompt consideration of an underlying neuromuscular cause. Careful neurologic examination for characteristic fatigable ocular and bulbar signs can help identify myasthenia gravis in such patients. Recognition of these clinical features is particularly important when initial symptoms are nonspecific or attributed to a metabolic disturbance. Early diagnosis and appropriate treatment of MG can reduce the risk of progression to respiratory complications. Clinicians should therefore maintain a high index of suspicion for coexisting neurologic disorders when weakness persists despite correction of an apparent reversible cause.

## References

[1] Gilhus NE (2016). Myasthenia gravis. N Engl J Med.

[2] Juel VC, Massey JM (2007). Myasthenia gravis. Orphanet J Rare Dis.

[3] Vincent A, Palace J, Hilton-Jones D (2001). Myasthenia gravis. Lancet.

[4] Thanvi BR, Lo TC (2004). Update on myasthenia gravis. Postgrad Med J.

[5] Keesey JC (2004). Clinical evaluation and management of myasthenia gravis. Muscle Nerve.

[6] Drachman DB (1994). Myasthenia gravis. N Engl J Med.

[7] Stedwell RE, Allen KM, Binder LS (1992). Hypokalemic paralyses: a review of the etiologies, pathophysiology, presentation, and therapy. Am J Emerg Med.

[8] Ignacio KH, Bagnas MA, Espiritu AI, Reyes JP (2019). Secondary hypokalemic paralysis with bulbar weakness and reversible electrophysiologic abnormalities: a case report and systematic review. J Clin Neurosci.

[9] Carr AS, Cardwell CR, McCarron PO, McConville J (2010). A systematic review of population based epidemiological studies in myasthenia gravis. BMC Neurol.

[10] Sanders DB, Wolfe GI, Benatar M (2016). International consensus guidance for management of myasthenia gravis: executive summary. Neurology.

[11] Narayanaswami P, Sanders DB, Wolfe G (2021). International consensus guidance for management of myasthenia gravis: 2020 update. Neurology.

[12] Jaretzki A 3rd, Barohn RJ, Ernstoff RM (2000). Myasthenia gravis: recommendations for clinical research standards. Ann Thorac Surg.

[13] Meriggioli MN, Sanders DB (2009). Autoimmune myasthenia gravis: emerging clinical and biological heterogeneity. Lancet Neurol.

[14] Schneider-Gold C, Gajdos P, Toyka KV, Hohlfeld RR (2005). Corticosteroids for myasthenia gravis. Cochrane Database Syst Rev.

[15] Palace J, Newsom-Davis J, Lecky B (1998). A randomized double-blind trial of prednisolone alone or with azathioprine in myasthenia gravis. Neurology.

[16] Romi F, Skeie GO, Aarli JA, Gilhus NE (2000). The severity of myasthenia gravis correlates with the serum concentration of titin and ryanodine receptor antibodies. Arch Neurol.

[17] Wolfe GI, Kaminski HJ, Aban IB (2016). Randomized trial of thymectomy in myasthenia gravis. N Engl J Med.

[18] Marx A, Chan JK, Coindre JM (2015). The 2015 World Health Organization classification of tumors of the thymus: continuity and changes. J Thorac Oncol.

[19] Engels EA (2010). Epidemiology of thymoma and associated malignancies. J Thorac Oncol.

